# The Genomic Signature of *Human Rhinoviruses A*, *B* and *C*

**DOI:** 10.1371/journal.pone.0044557

**Published:** 2012-09-13

**Authors:** Spyridon Megremis, Philippos Demetriou, Heidi Makrinioti, Alkistis E. Manoussaki, Nikolaos G. Papadopoulos

**Affiliations:** Allergy Department, 2nd Pediatric Clinic, University of Athens, Athens, Greece; Duke University School of Medicine, United States of America

## Abstract

Human rhinoviruses are single stranded positive sense RNA viruses that are presented in more than 50% of acute upper respiratory tract infections. Despite extensive studies on the genetic diversity of the virus, little is known about the forces driving it. In order to explain this diversity, many research groups have focused on protein sequence requirements for viable, functional and transmissible virus but have missed out an important aspect of viral evolution such as the genomic ontology of the virus. This study presents for the first time the genomic signature of 111 fully sequenced HRV strains from all three groups HRV-A, HRV-B and HRV-C. We observed an HRV genome tendency to eliminate CpG and UpA dinucleotides, coupling with over-representation of UpG and CpA. We propose a specific mechanism which describes how rapid changes in the HRV genomic sequence can take place under the strict control of conservation of the polypeptide backbone. Moreover, the distribution of the observed under- and over-represented dinucleotides along the HRV genome is presented. Distance matrice tables based on CpG and UpA odds ratios were constructed and viewed as heatmaps and distance trees. None of the suppressions can be attributed to codon usage or in RNA secondary structure requirements. Since viral recognition is dependent on RNA motifs rich in CpG and UpA, it is possible that the overall described genome evolution mechanism acts in order to protect the virus from host recognition.

## Introduction

Human rhinoviruses (HRVs) are non-enveloped, positive-sense, single stranded RNA viruses (+ssRNA) which belong to the genus *Enterovirus* in the family *Picornaviridae*. The HRV 3D-polymerase, necessary for synthesis of new genome, has no proof-reading capability a fact that estimates one to four mutations per replicative cycle for lytic RNA viruses, a number that would predict incredibly high nucleotide replacement rates for an RNA virus during an epidemic [1], [2]. However, the actual replacement rates are between 5×10^−2^ and 5×10^−4^ per site per annum [2], [3]. This presents an interesting case because in one hand RNA viruses can evolve rapidly, close to the maximum rate, compatible with maintaining genetic information, and with frequent recombination, yet their genomes are remarkably stable when grown under unchanging conditions [4]–[6]. The paradox between the predicted and observed replacement rates could be explained if effective selection processes were in operation during virus replication such as the strict control of functional RNA secondary structures, conservation of the polypeptide backbone and avoidance of “intolerable” nucleic acid immunostimulatory motifs and/or structures.

These viral evolutionary constraints can be depicted as anomalies in the occurrence of the sixteen dinucleotides (XpY) when comparing their odds ratios (R_XpY_). This dinucleotide bias estimator is usually referred to as the genomic signature or the dinucleotide odds ratio profile of a given sequence or genome of an organism. In order to better understand the evolutionary pressures responsible for the high HRV sequence variation observed amongst all fully sequenced HRV strains and how this is achieved we performed a dinucleotide odds ratio profiling analysis. We propose a novel evolutionary mechanism of their genomic sequences which is independent of codon usage and/or RNA structures but is controlled by the maintenance of a functional amino acid level equilibrium.

## Methods

### Data Acquisition

The complete genome sequences for 111 Human rhinovirus strains were downloaded from the National Center for Biotechnology Information (NCBI) website in GenBank (National Center for Biotechnology Information) format [7]. This reference set includes 75 *HRV-A* and 25 *HRV-B* serotypes. The *HRV-C* species has only recently been recognized, and up to date consists of 11 types whose complete genomes are known [8]–[12].

### Dinucleotide frequency analysis

The “Dinucleotide Properties Genome Browser (DiProGB)” (http://diprogb.fli-leibniz.de/) was used to generate sequence frequency statistics and to visualize nucleotide sequences as dinucleotide-encoded sequence graphs [13].

### Dinucleotide Odds Ratio Calculation

Dinucleotide odds ratio is the quotient of the probability of finding a dinucleotide in a given sequence divided by the product of the probabilities of finding each nucleotide that forms the pair in the same sequence, calculated as shown in Equation 1.

Equation 1: Calculation of dinucleotide odds ratio R_XpY_ for a single stranded sequence
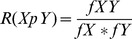



Dinucleotides with odds ratio values outside the 0.81–1.19 range were considered as having a low or high relative abundance, respectively, as proposed by Burge et al [14], [15].

### Sequence and structural alignment

RNA and protein sequence alignments were produced in the CLC RNA Free Workbench 4.4 (CLC bioA/S) and CLC Protein Free workbench 5.5.2 (CLC bio), respectively. Phylogenetic analysis based on sequence alignments were performed in the same platforms using neighbor joining algorithm and 100 bootstraps. Structural alignments were performed in the “Sequence to Structure (S2S)” package [16].

### Relative Informative Synonymous Codon Usage Calculation

Relative Synonymous Codon Usage Calculation (RSCU) is used to estimate codon bias for all codons which code for an amino acid with degeneracy greater than one. It is defined as the observed frequency of a codon *j* in a sequence *x* divided by the frequency expected *E* if all synonymous codons for the amino acid coded by *j* were equally frequent, as shown in Equation 2.

Equation 2: Calculation of RSCU
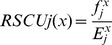



Expected values are calculated by counting the total number of synonymous codons for a given amino acid in the sequence divided by the number of existing codons that codes for it. Informative synonymous codons are defined as the trinucleotides containing a dinucleotide which is differentially represented in the odds ratio profiling (CpG, UpG, CpA and UpA) and encode for an amino acid which is also encoded by at least one trinucleotide without the aforementioned dinucleotides. Thus the non-informative codons UAC-UAT (Tyrosine), UGU-UGC (Cysteine), CAU-CAC (Histidine) and CAA-CAG (Glutamine) cannot be used in the analysis. All calculations were generated using the CALcal software [17].

### Pairwise distance analyses

**Figure 1:**
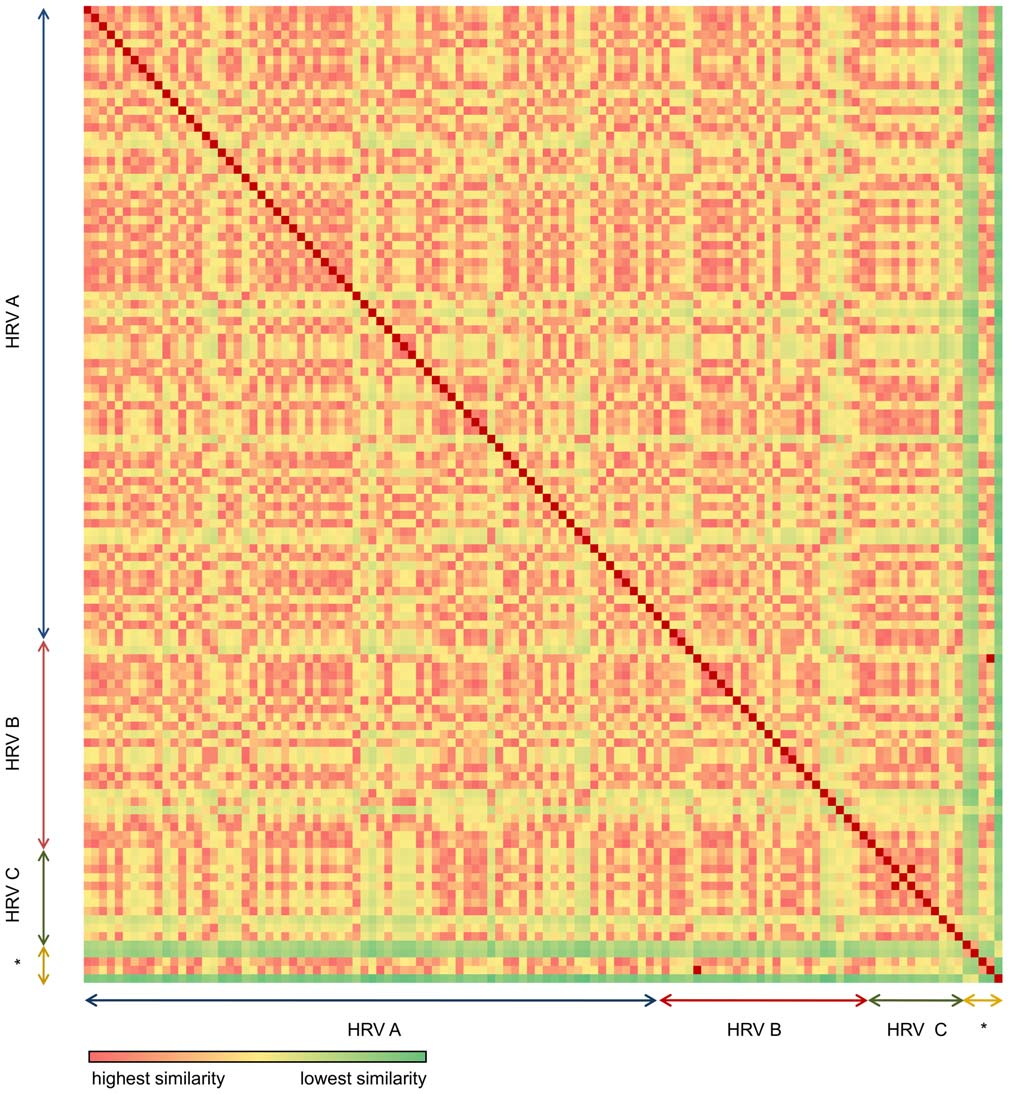
Heatmap of CpG odds ratio (midpoint 61). The difference of the odds ratios between strains is depicted in colours. The colour range used shows highest similarity with red, with decreasing similarity moving to yellow and least similarity in green. Asterisc: outgrouping of viruses with no CpG suppression (methods).

**Figure 2:**
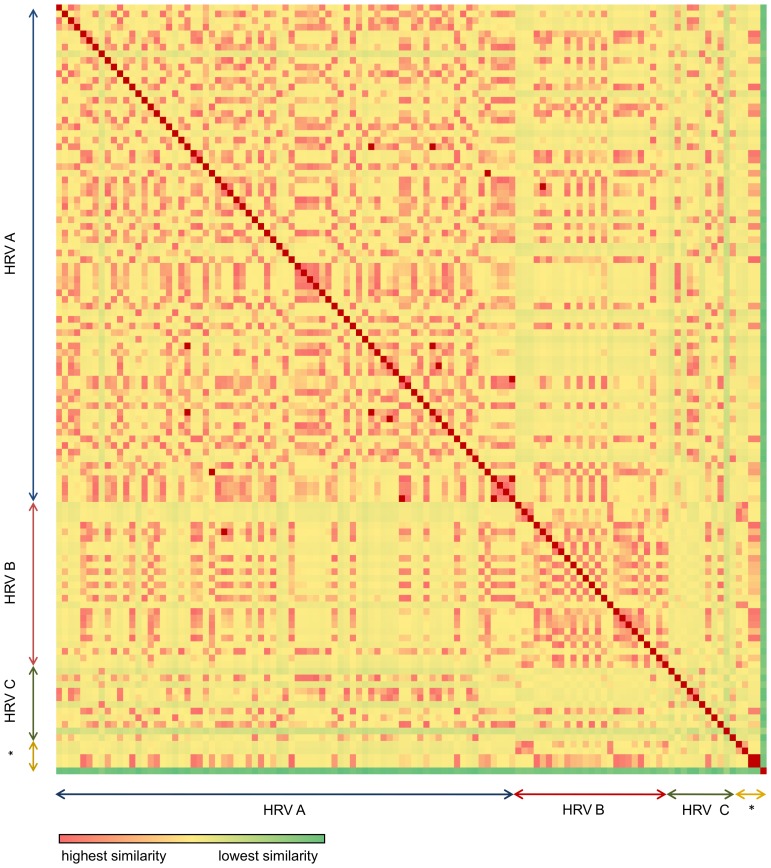
Heatmap of UpA odds ratio (midpoint 35). The difference of the odds ratios between strains is depicted in colours. The colour range used shows highest similarity with red, with decreasing similarity moving to yellow and least similarity in green. Asterisc: outgrouping of viruses with high UpA suppression (methods).

Matrices of pairwise distances based on odds ratios of CpG and UpA dinucleotides showing percentage differences between all pairs of the 111 HRV strains were constructed and presented as heatmaps in figures 1 and 2. These were further analysed using the PHYLIP package [18]. DRAWTREE and DRAWGRAM were used to visualize the results as pairwise distance trees (supplementary data, FS1, FS2). Neighbor Unweighted Pair Group Method with arithmetic mean (UPGMA) and Neighbor-Joining (NJ) algorithms were used to generate the best tree, along with the DAMBE software which utilizes the FastME method (used with default parameters) [19]. In all scenarios, CpG/UpA dinucleotide odds ratios of other single-stranded RNA viruses were included in the analysis based on the Rima and McFerran publication [15].

## Results

We analyzed the odds ratio R_XpY_ of the 16 dinucleotides for 111 HRV genomes comprising 75 HRV-A, 25 HRV-B and 11 HRV-C strains (Table S1, supplementary data). Mean R_XpY_ values along with minimum and maximum values are shown in table 1. We found three differentially represented dinucleotides (CpG, CpA, UpG) in the odds ratio profiling of all HRV sequences tested with p<0.001 (Dunns Repeated Measures test) along with a small borderline under-representation of UpA (mean R_UpA_: 0.82) which was consistent in most strains (range 0.69–0.90). The lowest R_UpA_ value was observed in HRV-QCE (0.69). CpA and UpG dinucleotides were highly over-represented in all human rhinovirus strains (Table 1). The highest R_UpG_ value was observed in HRV-QCE (1.52) which belongs to HRV-C. The highest R**_CpA_** value was found in HRV-A95 (1.51). The dinucleotide CpG was massively under-represented in all HRV strains (mean R_CpG_: 0.28). The highest CpG suppression was observed in HRV-A51, HRV-A81 and HRV-B84 (0.19). We also noticed a small over-representation of CpC in the HRV-A (1.22) and HRV-C (1.24) group and an even smaller over-representation of GpG only in HRV-C (1.20). None of the reversed dinucleotides (GpC, ApU, ApC, GpU) of the four differentially represented dinucleotides were over- or under-represented in our sequences indicating that the observed dinucleotide tendencies are not mononucleotide driven (table 1). CpG occurrence was significantly inverse correlated with C+G content (P<0.0001, r = 0.416) and the same applied for UpA/U+A content (P<0.0001, r = 0.362).

**Table 1:** 4×4 dinucleotide occurrence ratios showing mean R_XpY_ values in 111 Human rhinovirus strains.

HRV		Second base					
	First base		A	U	C	G	Row sum
	A	Mean	0.96	1.00	1.04	1.03	4.03
		95%c.l.	0.951–0.959	0.999–1.012	1.032–1.047	1.021–1.034	
		range	0.90–1.01	0.92–1.06	0.96–1.19	0.93–1.11	
	U	mean	0.82	1.02	0.86	**1.36**	4.06
		95%c.l.	0.812–0.823	1.012–1.028	0.851–0.865	1.356–1.375	
		range	0.69–0.90	0.94–1.13	0.75–0.96	1.26–1.52	
	C	mean	**1.35**	0.97	1.23	**0.26**	3.81
		95%c.l.	1.339–1.354	0.961–0.978	1.214–1.234	0.254–0.268	
		range	1.25–1.51	0.86–1.08	1.12–1.41	0.19–0.38	
	G	mean	0.96	0.98	0.92	1.16	4.02
		95%c.l.	0.958–0.968	0.976–0.990	0.911–0.934	1.147–1.166	
		range	0.89–1.02	0.80–1.07	0.79–1.09	1.05–1.37	
Column sum			4.09	3.97	4.05	3.81	

Dinucleotides with odds ratio values outside the 0.81–1.19 range were considered as having a low or high relative abundance, respectively, as proposed by Burge et al [14].

In order to understand the relationship of these discrepancies in the dinucleotide odds ratios we constructed a 4×4 occurrence table (table 1) [15]. In such a table both columns and rows of R_XpY_ values must sum to four since the expected R_XpY_ for all dinucleotides when no evolutionary pressure is present must equall one. If a bias of dinucleotide usage is arising by replacement of one nucleotide by another, a compensatory mechanism must maintain the four sum in both columns and rows. From this table it is obvious that CpG, UpA, UpG and CpA are all involved in a mechanism that acts in order to maintain a balance amongst the 16 dinucleotides. However, we observe that the level of CpG suppression is such that the four sum cannot be reached neither in the CpG containing column or row. From the same table can be seen that UpA even though only mildly under-represented is part of this mechanism. That is why we chose to include it in all of our subsequent analyses.

Next, we evaluated the distribution of the dinucleotide disturbances in the non-coding and coding regions of the human rhinovirus genome. The results are presented in table 2. The under-representation of CpG/UpA is higher in the coding regions coupled to the over-representation of CpA/UpG in all three HRV groups. This led us to investigate more our results. Table 3, shows the genomic distribution of the four mean R_XpY_ values in HRV-A, HRV-B and HRV-C in the non-coding 5′ and 3′ untranslated regions and in the four structural (capsid: VP4, VP2, VP3, VP1) and seven non-structural genes (2A^pro^, 2B, 2C, 3A, 3B, 3C^pro^, 3D^pol^). Surprisingly, in 5′UTR, R_CpG_ values are quite higher: 0.57 in HRV-A, 0.74 in HRV-B and 0.61 in HRV-C, suggesting considerably less suppression than in the rest of the genome. R_UpA_ values are 1.00 in HRV-A, 1.08 in HRV-B and 1.03 in HRV-C suggesting no suppression of the UpA dinucleotide. CpA is also not over-represented (HRV-A: 1.07, HRV-B: 0.96, HRV-C: 1.07), however R_UpG_ values seem to be constant around 1.31 (mean value). The highest UpA suppression is observed in the VP4 region (HRV-A: 0.65, HRV-B: 0.57, HRV-C: 0.65).

**Table 2:** Differentiated mean R_XpY_ values in genome sequence vs coding sequence.

		R_UpG_	R_CpA_	R_CpG_	R_UpA_
HRV-A	genome	1.36	1.33	0.25	0.85
	coding sequence	1.38	1.37	0.21	0.84
HRV-B	genome	1.32	1.37	0.27	0.81
	coding sequence	1.34	1.42	0.19	0.79
HRV-C	genome	1.43	1.34	0.30	0.82
	coding sequence	1.45	1.38	0.26	0.80

**Table 3:** Genomic distribution of differentiated mean R_XpY_ values in HRV A, B and C.

	HRV-A				HRV-B				HRV-C			
	R_UpA_	R_UpG_	R_CpA_	R_CpG_	R_UpA_	R_UpG_	R_CpA_	R_CpG_	R_UpA_	R_UpG_	R_CpA_	R_CpG_
5′UTR	1.00	*1.33*	1.07	**0.57**	1.08	*1.22*	0.96	**0.74**	1.03	*1.39*	1.07	**0.61**
VP4	***0.65***	1.09	1.43	0.32	***0.57***	1.24	1.56	0.34	***0.65***	1.29	1.43	0.37
VP2	0.87	1.39	1.47	0.23	0.82	1.47	1.52	0.23	0.86	1.43	1.46	0.32
VP3	0.89	1.40	1.49	0.18	0.86	1.35	1.50	0.15	0.83	1.40	1.54	0.31
VP1	0.84	1.48	1.48	0.20	0.80	1.40	1.40	0.21	0.76	1.41	1.39	0.23
2A	0.89	1.52	1.50	0.16	0.90	1.33	1.47	0.18	0.95	1.67	1.44	0.18
2B	0.88	1.20	1.39	0.14	0.76	1.24	1.54	0.11	0.85	1.36	1.57	0.28
2C	0.75	1.49	1.29	0.23	0.69	1.38	1.41	0.20	0.72	1.50	1.31	0.22
3A	1.05	1.28	1.23	0.13	0.94	1.36	1.36	0.06	0.92	1.21	1.34	0.17
3B	0.86	1.12	1.02	0.13	0.94	1.09	1.21	0.31	0.95	1.27	1.04	0.15
3C	0.86	1.30	1.35	0.15	0.83	1.22	1.41	0.25	0.79	1.60	1.45	0.27
3D	0.84	1.31	1.25	0.23	0.78	1.29	1.30	0.17	0.80	1.33	1.16	0.24
3′ UTR	0.97	0.88	0.85	0.00	0.98	0.67	0.86	0.00	1.05	1.35	0.77	0.00

R_CpG_ values printed in bold are highest (minimum suppression) in the 5′ UTR. Only R_UpG_ values printed in *italic* are kept in differentiated levels (R_XpY_>1.19) in 5′. R_UpA_ is highly suppressed in the VP4 region (bold and italic).

In order to investigate whether the observed dinucleotide tendencies are codon driven we compared them with RSCU values for all informative synonymous codons (table 4). In RSCU analysis if a certain amino acid is over- or under-represented in the protein sequence then its mean RSCU value should deviate significantly from the value 1. If this deviation correlates with the observed dinucleotide tendencies then a possible cause of these tendencies would be protein requirements for specific amino acids containing specific dinucleotides. In our RSCU table none of the amino acids has a mean RSCU value lower or higher than 1 suggesting that the observed dinucleotide suppressions and over-representations are not codon driven. More specifically the CpG dinucleotide encodes, as part of a coding trinucleotide, five different amino acids: Serine (S), Proline (P), Threonine (T), Alanine (A) and Arginine (A). One would expect that if CpG suppression is driven by codon usage the above amino acids would be represented with low mean RSCU values. Looking at the right side of the RSCU table it can be seen that the first eight codons with the lowest RSCU values contain CpGs. Alanine encoded by GCpG has an RSCU value ranging from 0.10 in HRV-A to 0.13 in HRV-B. However, alanine is also encoded by GCC (RSCU = 0.66–0.85), GCU (RSCU = 1.35–1.44) and GCpA (RSCU = 1.59–1.84). Threonine is encoded by ACpG (0.11–0.12), ACC (0.79–0.96), ACU (1.14–1.23) and ACpA (1.75–1.87). Serine is encoded by UCpG (0.12–0.17), AGC (0.54–0.70), UCC (0.65–0.78), UCU (1.07–1.16), AGU (1.50–1.55) and UCpA (1.73–2.04). Proline is encoded by CCpG (0.13–0.15), CCC (0.67–0.73), CCU (1.06–1.07) and CCpA (2.05–2.13). Finally, arginine is encoded by CpGG (0.13–0.24), CpGA (0.25–0.32), CpGU (0.36–0.60), CpGC (0.38–0.61) but also by AGG (1.38–1.44) and AGA (2.79–3.48). The above results suggest that the CpG suppression is not associated with specific amino acid usage since for every CpG-containing codon with a low RSCU value there are other informative synonymous codons with RSCU values well above 1, which compensate for the specific amino acid loss. Even in the case of arginine, which is known to be avoided in protein sequences, and is encoded by the most CpG-containing triplets (four versus one in the other amino acids), we observe a large compensation by AGG and AGA with the latter having the highest RSCU value in all HRV groups.

**Table 4:** Relative Synonymous Codon Usage Calculation (RSCU) values for all amino acids.

CODON	A.A	HRV-A	HRV-B	HRV-C	CODON	A.A	HRV-A	CODON	A.A	HRV-B	CODON	A.A	HRV-C
UUU	F	1.24	1.05	1.04	GCG	A	0.10	ACG	T	0.12	GCG	A	0.11
UUC	F	0.76	0.95	0.96	ACG	T	0.11	GCG	A	0.13	ACG	T	0.12
**UUA**	L	1.67	1.29	1.28	UCG	S	0.12	CCG	P	0.15	CCG	P	0.15
**UUG**	L	1.20	1.16	1.24	CCG	P	0.13	UCG	S	0.17	UCG	S	0.16
CUU	L	1.00	0.96	0.94	CGG	R	0.13	CGG	R	0.24	CGG	R	0.21
CUC	L	0.55	0.71	0.66	CGA	R	0.25	CGA	R	0.32	CGA	R	0.27
**CUA**	L	0.97	1.09	1.12	CGU	R	0.36	CGU	R	0.60	CGC	R	0.54
**CUG**	L	0.61	0.80	0.76	CGC	R	0.38	CGC	R	0.61	CGU	R	0.58
AUU	I	1.15	0.99	1.04	AGC	S	0.54	GGC	G	0.64	GGC	G	0.64
AUC	I	0.61	0.81	0.75	CUC	L	0.55	UGC	C	0.66	CUC	L	0.66
**AUA**	I	1.24	1.21	1.22	GGG	G	0.59	GGG	G	0.68	AGC	S	0.67
GUU	V	1.39	1.16	1.21	AUC	I	0.61	AGC	S	0.70	UGC	C	0.67
GUC	V	0.64	0.73	0.70	CUG	L	0.61	GAG	E	0.70	GGG	G	0.69
**GUA**	V	0.98	0.89	0.89	GAC	D	0.61	CUC	L	0.71	GUC	V	0.70
**GUG**	V	0.99	1.21	1.20	UGC	C	0.62	CCC	P	0.72	UCC	S	0.72
UCU	S	1.16	1.07	1.15	GGC	G	0.63	GUC	V	0.73	GAG	E	0.73
UCC	S	0.65	0.78	0.72	GUC	V	0.64	GAC	D	0.76	CCC	P	0.73
**UCA**	S	2.04	1.73	1.80	UCC	S	0.65	UCC	S	0.78	AUC	I	0.75
**UCG**	S	0.12	0.17	0.16	GCC	A	0.66	CUG	L	0.80	GAC	D	0.76
AGU	S	1.50	1.55	1.51	GAG	E	0.67	AAG	K	0.80	CUG	L	0.76
AGC	S	0.54	0.70	0.67	CCC	P	0.67	AUC	I	0.81	AAG	K	0.76
CCU	P	1.08	1.06	1.07	AAG	K	0.68	CAG	Q	0.81	CAG	Q	0.77
CCC	P	0.67	0.72	0.73	CAG	Q	0.71	GCC	A	0.85	GCC	A	0.85
**CCA**	P	2.13	2.07	2.05	UUC	F	0.76	GUA	V	0.89	GUA	V	0.89
**CCG**	P	0.13	0.15	0.15	AAC	N	0.76	UAC	Y	0.94	ACC	T	0.89
ACU	T	1.23	1.17	1.14	ACC	T	0.79	UUC	F	0.95	CUU	L	0.94
ACC	T	0.79	0.96	0.89	CAC	H	0.83	ACC	T	0.96	UAC	Y	0.96
**ACA**	T	1.87	1.75	1.85	UAC	Y	0.85	CUU	L	0.96	UUC	F	0.96
**ACG**	T	0.11	0.12	0.12	CUA	L	0.97	AAC	N	0.98	AAC	N	0.98
GCU	A	1.41	1.44	1.35	GUA	V	0.98	AUU	I	0.99	CAU	H	1.00
GCC	A	0.66	0.85	0.85	GUG	V	0.99	CAU	H	1.00	CAC	H	1.00
**GCA**	A	1.84	1.59	1.68	CUU	L	1.00	CAC	H	1.00	AAU	N	1.02
**GCG**	A	0.10	0.13	0.11	CCU	P	1.08	AAU	N	1.02	UUU	F	1.04
**UAU**	Y	1.15	1.06	1.04	UAU	Y	1.15	UUU	F	1.05	AUU	I	1.04
**UAC**	Y	0.85	0.94	0.96	AUU	I	1.15	CCU	P	1.06	UAU	Y	1.04
**CAU**	H	1.17	1.00	1.00	UCU	S	1.16	UAU	Y	1.06	CCU	P	1.07
**CAC**	H	0.83	1.00	1.00	CAU	H	1.17	UCU	S	1.07	CUA	L	1.12
**CAA**	Q	1.29	1.19	1.23	UUG	L	1.20	CUA	L	1.09	ACU	T	1.14
**CAG**	Q	0.71	0.81	0.77	ACU	T	1.23	UUG	L	1.16	UCU	S	1.15
AAU	N	1.24	1.02	1.02	AAU	N	1.24	GUU	V	1.16	GUG	V	1.20
AAC	N	0.76	0.98	0.98	AUA	I	1.24	ACU	T	1.17	GUU	V	1.21
AAA	K	1.32	1.20	1.24	UUU	F	1.24	CAA	Q	1.19	AUA	I	1.22
AAG	K	0.68	0.80	0.76	CAA	Q	1.29	AAA	K	1.20	CAA	Q	1.23
GAU	D	1.39	1.24	1.24	GGA	G	1.29	GGA	G	1.20	AAA	K	1.24
GAC	D	0.61	0.76	0.76	AAA	K	1.32	AUA	I	1.21	GAU	D	1.24
GAA	E	1.33	1.30	1.27	GAA	E	1.33	GUG	V	1.21	UUG	L	1.24
GAG	E	0.67	0.70	0.73	UGU	C	1.38	GAU	D	1.24	GAA	E	1.27
**UGU**	C	1.38	1.34	1.33	GAU	D	1.39	UUA	L	1.29	GGA	G	1.27
**UGC**	C	0.62	0.66	0.67	GUU	V	1.39	GAA	E	1.30	UUA	L	1.28
**CGU**	R	0.36	0.60	0.58	AGG	R	1.40	UGU	C	1.34	UGU	C	1.33
**CGC**	R	0.38	0.61	0.54	GCU	A	1.41	GCU	A	1.44	GCU	A	1.35
**CGA**	R	0.25	0.32	0.27	GGU	G	1.49	AGG	R	1.44	AGG	R	1.38
**CGG**	R	0.13	0.24	0.21	AGU	S	1.50	GGU	G	1.48	GGU	G	1.41
AGA	R	3.48	2.79	3.02	UUA	L	1.67	AGU	S	1.55	AGU	S	1.51
AGG	R	1.40	1.44	1.38	GCA	A	1.84	GCA	A	1.59	GCA	A	1.68
GGU	G	1.49	1.48	1.41	ACA	T	1.87	UCA	S	1.73	UCA	S	1.80
GGC	G	0.63	0.64	0.64	UCA	S	2.04	ACA	T	1.75	ACA	T	1.85
GGA	G	1.29	1.20	1.27	CCA	P	2.13	CCA	P	2.07	CCA	P	2.05
GGG	G	0.59	0.68	0.69	AGA	R	3.48	AGA	R	2.79	AGA	R	3.02

Left side: RSCU values grouping based on codons. Right side: Codon grouping based on increasing RSCU values. Bold: informative codons.

The UpA dinucleotide is borderline under-represented in the HRV-B (R_UpA_ = 0.81) and less in HRV-C (R_UpA_ = 0.82) and HRV-A (R_UpA_ = 0.85). However, the UpA-containing codons are not under-represented in the RSCU table; leucine (L) and valine (V) can be encoded by the UUpA (RSCU = 1.28–1.67), CUpA (0.97–1.12), UUpG (1.16–1.24), CUpG (0.61–0.76), CUU, CUC and GUpA (0.89–0.98), GUpG (0.99–1.21), respectively. Isoleucine (I) encoded by AUpA has RSCU values ranging between 1.21 and 1.24 and is not under-represented. Furthermore in the left side of the RSCU table 50% of the UpA-containing informative codons in HRV-A and 75% in HRV-B and HRV-C are grouped in the region where RSCU values are >1. Even the non-informative UpAC-UpAU (Tyrosine) codons have a mean RSCU value of 1. The above results suggest that UpA suppression is not driven by codon choice.

The dinucleotide CpA which is over-represented in the odds ratio profiling “encodes” serine (UCpA), proline (CCpA), threonine (ACpA), alanine (GCpA), histidine (CpAU-CpAC) and glutamine (CpAA-CpAG). The last two are non-informative synonymous codons. In the RSCU table the CpA-containing codons for A, T, S and P are located on the bottom right side where the highest RSCU values are located and as discussed above are also encoded by CpG-containing trinucleotides. The results suggest that the observed increase in CpA levels have a same impact in the CpA “encoded” aminoacids which are also increased. Since the same amino acids can also be encoded by CpG containing codons these data suggest that the high R_CpA_ values can be attributed to the need of the protein sequence to equilibrate the “loss” of the CpG-encoded amino acids by a CpG>CpA (G>A) transition.

On the other hand, UpG over-representation was the highest amongst all dinucleotides in all HRVs and we expected to find a similar over-representation of the UpG “encoded” amino acids as in the case of CpAs. However, only 25% in HRV-A (RSCU = 1.20) and 50% in HRV B and C (1.16–1.34) of the informative UpG-containing codons have RSCU values>1. These results generate an important question: How HRV maintains low levels of UpG “encoded” amino acids while having high numbers of UpGs (mean HRV R_UpG_ = 1.38) in its coding sequence (reaches 1.67 in 2A region)? One possible way for a coding nucleotide sequence to adjust dinucleotide frequencies without affecting (or partly affecting) the encoded amino acids is by incorporating the dinucleotide in codon junctions. Although codon usage fixes the frequency with which a dinucleotide is present in positions 1 and 2 and in positions 2 and 3, it has no effect on the incidence of junctional XpY dinucleotides, in which the mononucleotide X occupies the third position of a codon and Y the first position of the following codon. Table 5 shows the distribution of the 4 differentially represented in the odds ratio profiling dinucleotides in codons and in codon junctions. As expected, in all HRVs more UpGs occupy positions in codon junctions than in codons (57.5% versus 42.5%, respectively) a percentage reaching 60% in HRV A! Since UpG and UpA containing codons encode for the same aminoacids, these data suggest a UpA>UpG (A>G) transition. Furthermore, UpA is the second most abundant dinucleotide in codon junction position (45.5%) indicating a direct relationship with UpG.

**Table 5:** Distribution of over- and under-represented dinucleotides in codons and codon junctions (counts and percentages).

	UpG		CpG		CpA		UpA	
	codon	junction	codon	junction	codon	junction	codon	junction
HRV	208.72	282	29.35	21.38	378.55	188.35	273.22	227.9
	42.5%	57.5%	57.8%	42.2%	66.8%	33.2%	54.5%	45.5%
HRV-A	202.52	295	29.01	18.88	372.72	174.48	276.72	241.9
	40.7%	59.3%	60.6%	39.4%	68.1%	31.9%	53.3%	46.7%
HRV-B	218.08	249.24	24.88	24.32	402.12	206.92	271.32	202.92
	46.6%	53.4%	50.6%	49.4%	66%	34%	57.2%	42.8%
HRV-C	231.8	266.3	43.00	32.8	363.4	245.90	251.8	185.1
	46.5%	53.5%	56.7%	43.3%	60%	40%	57.63%	42.37%

Up to this point a CpG>CpA and UpA>UpG transition mechanism has been established which is guided by HRV aminoacid balance but is not driven by codon usage. However when looking at the distribution of the odds ratio values for CpG, UpA, UpG and CpA in the non coding versus the coding region of the genome we observed that in the 5′UTR CpG suppression can only be coupled with UpG over-representation suggesting a CpG>UpG (C>U) transition. This has a dual implication for our results: (1) The input in UpG increase originates also from CpG suppression apart from UpA suppression, however because UpGs are mostly located in codon junctions this has a little effect in the encoded UpG-amino acids, (2) UpA suppression is probably masked by CpG>CpA and CpG>UpG which predicts increased numbers of UpAs and not decreased as in our case.

In order to visualize the depth and variation of CpG/UpA suppressions amongst HRV strains, we constructed color-coded matrices of pairwise distances showing percentage differences between all pairs of HRV strains (Figures 1 and 2). We also draw pairwise distance trees based on the odds ratio values of the two under-represented dinucleotides CpG/UpA (FS1 and FS2, supplementary data). These trees do not imply phylogenetic relationships between the strains.

## Discussion

Even though the sequence and structure similarity of HRVs have been extensively investigated, this is the first study that presents the genomic signature of Human rhinoviruses and proposes a possible evolutionary mechanism of their genomic sequences. Our analysis revealed a CpG/UpA under- and CpA/UpG over-representation in all of 111 HRV genomic sequences. CpG/C+G and UpA/U+A are inverse correlated. The consequence of this inverse correlation is that when the expected number of CpG/UpA is low due to a low C+G/U+A content, the observed numbers are further suppressed. The under-representation of CpG/UpA is higher in the coding regions coupling with the over-representation of CpA/UpG in all three HRV groups. RSCU analysis suggests that none of the observed suppression tendencies are codon driven but the dinucleotide transitions are most probably determined by the HRV amino acid functional balance. We used RSCU analysis to investigate how this suppression/over-representation mechanism acts since the encoded polyprotein has a pivotal role in “specifying” the genomic sequence, and no mechanism can act ignoring requirements for the translation of specific amino acids. This fact has been missed in most publications concerning the genomic signature of various ssRNA viruses.

Based on our observations we propose a possible mechanism of HRV genome evolution: (1) A CpG suppression by transition of CpG>CpA (G>A) and CpG>UpG (C>U) takes place leading to (2) a subsequent increase in CpA and UpG numbers, with the latest being the highest. Interestingly, it seems that increased CpA-containing codons act in order to restore balance in the decrease of the amino acids encoded by CpG-containing synonymous codons. This is further supported by the fact that in the non-coding 5′UTR where CpG suppression is minimum there is no over-representation of CpA, suggesting a possible mechanism where the CpG>CpA transition is “active” mostly or only at the coding region of the genome. An interesting finding is that while a UpG increase would “normally” be depicted in subsequent increase of the amino acids encoded by UpG-containing codons, this is kept to a minimum by the localization of UpGs in codon junction positions in higher percentage than in codon positions in the coding sequence. The fact that in the 5′ UTR where the UpG over-representation can only be coupled with the CpG suppression suggests a CpG>UpG transition, (3) there is an initial UpA suppression also leading to a compensatory increase in UpG by transition of UpA>UpG (A>G). This is masked to a degree by the CpG suppression mechanism. Furthermore, UpG containing codons are synonymous with UpA containing codons. Overall, this highly sophisticated process ensures that effective suppression of CpG/UpA can take place without altering a functional balance in the amino acids encoded by the over-or-under-represented dinucleotide-containing codons. By this way it seems possible that the HRV genomic sequence can change in a “sub-protein” level. This is justified by the fact that the protein sequence of the virus which is mainly determined by viral (structural and non-structural proteins) and host (receptor utilization such as ICAM1 and LDL-R) structural requirements needs to be conserved amongst different strains thus no dramatic changes in the amino acid sequence can take place. However, the dinucleotide frequencies can change without affecting the encoded polyprotein. Possible reasons that could lead to CpG/UpA suppression are discussed below.

Karlin et al suggested that a potential reason for CpG suppression was the enhanced free energy for CpGs in double-stranded RNA [20]. Rima et al successfully argued that the substitutions of CpG by CpA/UpG or UpA by UpG are unlikely to affect the overall stability of any complementary or double-stranded intermediate, thus this mechanism cannot be applied in RNA viruses [15]. This is also evidenced in our results. The 5′UTR contains the internal ribosome entry site complex which generates the main replication signal of HRV. The IRES contains six stem–loop subdomains and the UA-rich polypyrimidine tract. If Karlin's suggestion applied for Human rhinovirus then we would observe higher suppression of CpGs and UpAs in the 5′UTR than in the rest of the genome since the IRES is a region rich in RNA secondary structures. However, we observe the least CpG suppression and no UpA suppression than in the rest of the genome. Furthermore, in our RNA secondary structure alignments we did not observe any CpG under-representation in the specific structures (data not shown). Additionally, it is known that viral RNA genomes that form complex and extensive secondary structures through internal base-pairing are tightly evolutionary constrained since based substitutions in any of the multiple sites that interact to form the structure require matching substitutions elsewhere such that the stem-loops are conserved [21]. On the other hand base-pair mismatches in RNA secondary structures can provide flexibility needed for conformational changes to take place. In vertebrate DNA genomes, methylated cytosines are prone to mutate through spontaneous deamination, generating the TpG with a mismatch pair T/G. This mismatch will in turn cause a mutation in the opposite strand if replication occurs without repair, leading to the appearance of the dinucleotide CpA as well. The methylation of genomes of RNA viruses without a DNA intermediate has not been studied, therefore cytosine methylation at this point cannot be considered a potential reason for the observed CpG suppression. In any case, if CpG is a hot spot of mutation with deleterious functional or structural consequences for the proteins, a reduction in CpG occurrence would be selectively favorable for virus viability.

UpA dinucleotide suppression could be a mosaic of numerous reasons: (i) UpAs are avoided in genomic sequences since they participate in the trinucleotides that encode stop codons. By reducing UpAs inside the genome, the possibility of generating deleterious for the protein sequence stop codons is further minimized. (ii) Beutler and colleagues suggested that a potential reason for UpA suppression is the susceptibility of UpA to RNase activity. They argued that the suppression of TpA in DNA is caused by the instability of UpA in RNA as the suppression was greater in exons than in introns and non-transcribed regions [22]. This is also evidenced in our results were there is a 0.20–0.30 difference in R_UpA_ between the non-coding and the coding sequence of HRV.

It is known that CpG when unmethylated in a DNA sequence can induce a strong immunostimulatory response on mammalian immune cells [23]. This is triggered by the intracellular pattern recognition receptor (PRR) Toll-like 9 (TLR9) which recognizes CpG-unmethylated DNA and triggers several immune responses [24]. Since the vertebrate immune system relies on non-methylated CpG recognition as a sign of infection and the observed CpG under-representation is present only in vertebrate viruses, it is reasonable to suggest that a similar mechanism with TLR9 could be used in RNA viruses [25]. Greenbaum and colleagues observed an inverse correlation of CpG depletion and C+G content in vertebrate infecting viruses with no such bias found in viruses with high C+G content [26]. Lobo and colleagues noticed a very similar CpG depletion tendency in vertebrate genes as well, suggesting that the genomes of RNA vertebrate viruses are selected to mimic some features of host mRNA to avoid immune system detection with a still unknown anti-viral mechanism [25]. Furthermore, it has been shown that ssRNA with unmethylated CpG motifs can stimulate CD14^+^CD11c^+^ monocytes to produce IL-12. These CpG oligoribonucleotides can also stimulate PBMCs to activate NF-κB and p38 MAPK. The activation of cells is not mediated by any known dsRNA, ssRNA or dsRNA receptor, but is abrogated if the 5′ position of C becomes methylated, similar to that of CpG DNA [27]. Furthermore, in a recent publication by Jimenez-Baranda, it was shown that CpG motifs in a UA-rich context quantitatively control pDC activation in Influenza virus infections [28]. The above suggest that CpG suppression in the HRV genome can be a counter-defense mechanism to escape RNA CpG motif recognition by the immune system.

Recently, Forsbach and colleagues identified single stranded RNA sequences containing specific sequence motifs that preferentially activate human TLR8- or TLR7/8-mediated signaling. The authors defined specific GU-rich 4-mer sequences, activating human TLR7/8 by inducing IFN-α and pro-inflammatory cytokines and chemokines from cells expressing only TLR7 or TLR7/8. They also defined AU-rich 4-mers like UAUA, AUAU, AUAC, UAUU and UUAU which were able to induce TNF-α production [29]. Up to date GU-containing RNA sequences can be found in the 3′ genome of –ssRNA viruses of the order *Mononegavirales* including *vesicular stomatitis virus*, *Sendai virus*, *Human respiratory syncytial virus* (RSV) and *influenza virus*. Th1 cytokines are involved in the immune response to negative strand viruses via TLR8 and can be found upon stimulation of human PBMCs with GU rich ORNs derived from these sequences [30]–[32]. In contrast *alphaviruses* with a +ssRNA genome which contain conserved sequence elements in the 3′ UTR with AU rich 4-mer motifs cause Th2 cytokines production in the central nervous system upon experimental allergic encephalomyelitis infection [33]. It seems that the innate immune system may have evolved to recognize specific pathogen RNA regions, resulting in differential patterns of immune responses giving rise to prevalent Th1 or Th2 responses. These responses may be at least in part driven by TLRs, leading to innate responses with strong or weak type I IFN-dependent cytokine and chemokine production. Furthermore, it is known that RNA sensing in the 2–5A pathway is performed by the OAS family of proteins. Specifically, the activation of the OAS1 gene requires in vitro transcribed RNA to generate 2–5A. Recently, the consensus sequence nnWWnnnnnnnnnWGn (W:U or A) was demonstrated to be important for OAS1 activation [34]. Indeed, a UpA recognition system in viral RNA sequences has been described as a vertebrate immune response mechanism. In *Flaviviridae* family, *West Nile Virus* (WNV) and *Hepacivirus* are known to be recognized by RNase L, preferentially at UpA or UpU sites [35], [36]. From the above it seems possible that viral-recognition strategies by the immune system could drive human rhinovirus UpA suppression.

We used CpG/UpA odds ratio values to compare 111 HRV genomic sequences. The foot and mouth disease virus, the Coxsackievirus and the Poliovirus type 1 were used for out-grouping based on published CpG/UpA odds ratios (methods). The results can be viewed as heatmaps in figures 1 and 2 and as pairwise distance trees in figures S1 and S2 in supplementary data. HRV-N10 with the highest R_CpG_ (less suppressed) clustered on its own (figure S1, supplementary data). HRV-QCE with the lowest R_UpA_ (higher suppression) also clustered on its own (figure S2, supplementary data). An important difference between CpG and UpA heatmaps is the considerably higher UpA content variation amongst all strains in the 3 different groups but also in each group. On the other hand, we observe a more even distribution of CpG suppression in the three groups. This could have an implication regarding the capability of each strain to further suppress CpGs and UpAs. Furthermore we included in our analysis HRV-A13F03 (FJ445117) and HRV-A54F05 (FJ445139) to show that strains with high percentage similarity in sequence and protein alignment can cluster in different groups based on the CpG odds ratio measure (figure S1, supplementary data). For example, the genomes of HRV-A13ATCC (R_CpG_: 0.25, R_UpA_: 0.83) and HRV-A13F03 (R_CpG_: 0.29, R_UpA_: 0.82) are different by 530 bases (7.42%). 90% of these differences involve changes in CpN, NpG, UpN and NpA (data not shown).

This work provides a foundation for understanding HRV genomic diversity and proposes essential factors that may influence HRV fitness and evolution. The genomic signature of Human rhinoviruses A, B and C is being presented in this study for the first time. All HRVs show CpG/UpA suppression. The CpG suppression is counteracted by the over-representation of CpA/UpG. The highest CpG suppression is observed in the coding region of the genome. Furthermore, CpA-containing codons seem to function as “balancers” for the decrease of CpG-containing codons in the coding region of the genome, implying region-specificity for CpG>CpA transition. UpGs are uniformly over-represented in the whole genome. Their increase can also be attributed to the UpA>UpG transition. UpGs are largely located in codon junction positions providing HRV with a useful tool for altering CpG/UpA numbers in the coding sequence without affecting the encoded amino acid. UpA is mildly under-represented in the coding sequence of HRV, however we believe that the real suppression is higher but is masked by the CpG suppression mechanism. None of the suppressions can be attributed to codon usage of the HRV protein or in structural requirements, suggesting other possible evolutionary pressures that could be attributed to the avoidance of viral-recognition by the innate immune system. However, the role of CpG/UpA dinucleotides in the infectious cycle of various HRV strains has to be experimentally proven. It would also be fascinating to identify specific patterns/motifs containing CpG/UpA in the genome sequence of HRV. Additionally, codon deoptimization with concurrent increases in the frequencies of CpG and UpA dinucleotides in RNA virus genomes may provide a novel general approach to the design of vaccines with stable genetic propreties [37]. Finally, HRV clustering based on the odds ratio values of the suppressed CpG/UpA dinucleotides may prove to be a useful tool to understand “immune-driven” selection pressures that act on the HRV genome sequences. However, a basic implication of our analysis is that the genomic ontology of each HRV strain can play an important role in various aspects of viral recognition and should be carefully examined.

## Supporting Information

Table S1 **R_XpY_ values for all 16 dinucleotides in 111 HRV full genome sequences.** The X and Y mononucleotides are the first and second bases of any XpY dinucleotide, respectively. Values <0,81 or >1,19 are printed in bold.
(DOCX)

Figure S1 **Pairwise distance tree based on R_CpG_ values constructed using the FastME algorithm.**
(TIF)

Figure S2 **Pairwise distance tree based on R_UpA_ values values constructed using the FastME algorithm.**
(TIF)
