# Supplementary material for: The Genomic Signature of Human Rhinoviruses A, B and C
Source: PLoS One. 2012 Sep 13;7(9):e44557. doi: 10.1371/journal.pone.0044557 (PMC3441561; doi:10.1371/journal.pone.0044557)
Supplement: Table S1 — RXpY values for all 16 dinucleotides in 111 HRV full genome sequences. The X and Y mononucleotides are the first and second bases of any XpY dinucleotide, respectively. Values <0,81 or >1,19 are printed in bold. (DOCX) [file pone.0044557.s001.docx]

**Table S1: R_XpY_values for all 16 dinucleotides in 111 HRV full genome sequences**. The X and Y mononucleotides are the first and second bases of any XpY dinucleotide, respectively. Values <0,81 or >1,19 are printed in bold

|  | **X nucleotide** | **A** | | | | **C** | | | | **G** | | | | **U** | | | |
| --- | --- | --- | --- | --- | --- | --- | --- | --- | --- | --- | --- | --- | --- | --- | --- | --- | --- |
| No | **Y nucleotide** | **A** | **C** | **G** | **U** | **A** | **C** | **G** | **U** | **A** | **C** | **G** | **U** | **A** | **C** | **G** | **U** |
|  | **HRV strains** |  |  |  |  |  |  |  |  |  |  |  |  |  |  |  |  |
| 1 | 1b | 0.97 | 1.04 | 1.02 | 1.00 | **1.31** | 1.18 | **0.25** | 1.03 | 0.95 | 0.94 | 1.14 | 1.00 | 0.88 | 0.88 | **1.35** | 0.98 |
| 2 | 29 | 0.98 | 1.01 | 1.01 | 1.01 | **1.33** | **1.21** | **0.26** | 1.00 | 0.95 | 0.96 | 1.17 | 0.97 | 0.85 | 0.89 | **1.34** | 1.01 |
| 3 | 2 | 0.95 | 1.05 | 0.98 | 1.04 | **1.32** | **1.24** | **0.28** | 0.98 | 0.96 | 0.99 | 1.16 | 0.95 | 0.88 | **0.79** | **1.40** | 1.00 |
| 4 | 1 | 0.98 | 1.03 | 0.98 | 1.02 | **1.32** | **1.21** | **0.24** | 1.00 | 0.95 | 0.95 | 1.16 | 0.98 | 0.87 | 0.86 | **1.38** | 0.98 |
| 5 | 10 | 0.98 | 1.03 | 1.01 | 1.00 | **1.36** | 1.17 | **0.29** | 0.97 | 0.95 | 0.97 | 1.12 | 1.00 | 0.82 | 0.87 | **1.39** | 1.02 |
| 6 | 100 | 0.98 | 1.04 | 0.97 | 1.02 | **1.36** | **1.22** | **0.24** | 0.98 | 0.95 | 1.02 | 1.13 | 0.96 | 0.83 | 0.81 | **1.43** | 1.02 |
| 7 | 11 | 0.96 | 0.99 | 1.02 | 1.04 | **1.33** | **1.22** | **0.22** | 1.01 | 0.99 | 1.00 | 1.08 | 0.96 | 0.84 | 0.87 | **1.42** | 0.97 |
| 8 | 12 | 1.00 | 1.02 | 1.06 | 0.95 | **1.35** | 1.19 | **0.23** | 1.01 | 1.00 | 0.96 | 1.06 | 0.98 | **0.76** | 0.87 | **1.42** | 1.07 |
| 9 | 13 | 0.97 | 1.03 | 0.99 | 1.03 | **1.31** | **1.24** | **0.25** | 1.01 | 1.01 | 0.96 | 1.13 | 0.93 | 0.83 | 0.84 | **1.42** | 1.00 |
| 10 | 15 | 0.97 | 1.04 | 1.00 | 1.01 | **1.31** | **1.31** | **0.28** | 0.95 | 0.98 | 0.89 | 1.12 | 1.00 | 0.85 | 0.83 | **1.38** | 1.02 |
| 11 | 16 | 0.92 | 1.06 | 1.04 | 1.02 | **1.37** | 1.19 | **0.21** | 0.99 | 0.96 | 0.96 | 1.12 | 0.99 | 0.88 | 0.83 | **1.39** | 0.99 |
| 12 | 18 | 0.94 | 1.01 | 1.03 | 1.05 | **1.36** | **1.26** | **0.24** | 0.93 | 0.98 | 0.96 | 1.15 | 0.94 | 0.85 | 0.83 | **1.37** | 1.03 |
| 13 | 19 | 0.99 | 0.98 | 1.00 | 1.02 | **1.31** | **1.23** | **0.29** | 0.99 | 0.98 | 1.01 | 1.14 | 0.92 | 0.82 | 0.87 | **1.36** | 1.03 |
| 14 | 20 | 0.91 | 1.06 | 1.04 | 1.05 | **1.37** | **1.21** | **0.23** | 0.94 | 0.94 | 0.89 | **1.24** | 0.98 | 0.90 | 0.86 | **1.32** | 1.00 |
| 15 | 21 | 0.99 | 1.00 | 1.00 | 1.02 | **1.33** | **1.25** | **0.25** | 0.97 | 0.94 | 0.95 | 1.17 | 0.98 | 0.84 | 0.87 | **1.37** | 1.01 |
| 16 | 22 | 1.00 | 1.05 | 0.97 | 0.98 | **1.33** | 1.15 | **0.30** | 1.02 | 0.94 | 0.96 | 1.18 | 0.96 | 0.81 | 0.86 | **1.39** | 1.03 |
| 17 | 23 | 0.99 | 1.02 | 0.97 | 1.02 | **1.31** | 1.16 | **0.33** | 1.00 | 0.98 | 0.99 | 1.11 | 0.96 | 0.83 | 0.88 | **1.40** | 1.00 |
| 18 | 24 | 0.96 | 1.02 | 1.04 | 1.01 | **1.34** | **1.23** | **0.22** | 0.99 | 0.98 | 0.96 | 1.10 | 0.98 | 0.85 | 0.85 | **1.38** | 1.01 |
| 19 | 25 | 0.96 | 1.02 | 1.03 | 1.01 | **1.31** | **1.33** | **0.24** | 0.96 | 0.99 | 1.00 | 1.08 | 0.96 | 0.87 | **0.77** | **1.38** | 1.04 |
| 20 | 28 | 0.93 | 1.01 | 1.07 | 1.02 | **1.31** | **1.25** | **0.23** | 1.00 | 0.95 | 0.95 | 1.15 | 0.99 | 0.90 | 0.85 | **1.35** | 0.98 |
| 21 | 30 | 0.97 | 1.00 | 0.98 | 1.05 | **1.31** | **1.21** | **0.32** | 0.98 | 0.97 | 0.97 | 1.15 | 0.95 | 0.85 | 0.88 | **1.37** | 0.99 |
| 22 | 32 | 0.97 | 1.05 | 0.98 | 1.02 | **1.32** | **1.30** | **0.28** | 0.94 | 0.95 | 0.94 | 1.16 | 0.99 | 0.87 | **0.80** | **1.37** | 1.02 |
| 23 | 33 | 0.96 | 1.00 | 1.04 | 1.01 | **1.36** | **1.21** | **0.22** | 0.99 | 0.98 | 1.00 | 1.05 | 0.99 | 0.82 | 0.85 | **1.43** | 1.00 |
| 24 | 34 | 0.99 | 1.06 | 0.95 | 1.01 | **1.34** | **1.28** | **0.27** | 0.93 | 0.96 | 0.93 | 1.16 | 0.99 | 0.82 | **0.80** | **1.43** | 1.04 |
| 25 | 36 | 0.99 | 0.97 | 1.03 | 1.01 | **1.25** | **1.34** | **0.24** | 1.01 | 0.97 | 0.93 | 1.14 | 0.98 | 0.86 | 0.85 | **1.39** | 0.99 |
| 26 | 38 | 0.97 | 1.00 | 0.99 | 1.04 | **1.38** | **1.20** | **0.28** | 0.93 | 0.96 | 0.90 | 1.16 | 1.00 | 0.81 | 0.94 | **1.37** | 1.00 |
| 27 | 39 | 0.95 | 1.06 | 1.02 | 1.01 | **1.34** | 1.15 | **0.25** | 1.03 | 0.98 | 0.99 | 1.12 | 0.95 | 0.84 | 0.84 | **1.40** | 1.01 |
| 28 | 40 | 0.95 | 0.99 | 1.03 | 1.05 | **1.38** | **1.23** | **0.22** | 0.95 | 0.98 | 1.02 | 1.12 | 0.93 | 0.83 | 0.86 | **1.38** | 1.03 |
| 29 | 41 | 0.95 | 1.04 | 0.98 | 1.04 | **1.34** | **1.21** | **0.26** | 1.00 | 0.99 | 0.91 | 1.16 | 0.96 | 0.83 | 0.88 | **1.41** | 0.98 |
| 30 | 43 | 0.96 | 0.97 | 1.01 | 1.06 | **1.37** | **1.25** | **0.23** | 0.95 | 0.94 | 1.05 | 1.14 | 0.80 | 0.86 | 0.84 | **1.38** | 1.00 |
| 31 | 44 | 0.96 | 1.02 | 1.01 | 1.03 | **1.33** | **1.20** | **0.26** | 1.01 | 0.98 | 1.00 | 1.15 | 0.92 | 0.85 | 0.85 | **1.35** | 1.02 |
| 32 | 45 | 0.93 | 1.04 | 1.08 | 1.00 | **1.38** | **1.23** | **0.25** | 0.92 | 0.98 | 0.89 | 1.16 | 1.00 | 0.83 | 0.87 | **1.33** | 1.06 |
| 33 | 46 | 0.94 | 1.02 | 1.04 | 1.04 | **1.32** | **1.22** | **0.24** | 1.00 | 0.96 | 0.92 | **1.20** | 0.97 | 0.89 | 0.89 | **1.33** | 0.97 |
| 34 | 47 | 0.98 | 0.96 | 1.03 | 1.03 | **1.42** | **1.23** | **0.26** | 0.90 | 0.92 | 1.09 | 1.11 | 0.96 | 0.83 | 0.85 | **1.34** | 1.05 |
| 35 | 49 | 0.98 | 1.04 | 0.97 | 1.02 | **1.31** | 1.14 | **0.32** | 1.01 | 0.95 | 1.00 | 1.16 | 0.95 | 0.85 | 0.86 | **1.38** | 1.00 |
| 36 | 50 | 0.97 | 0.99 | 1.01 | 1.03 | **1.32** | **1.29** | **0.22** | 0.97 | 0.98 | 0.93 | 1.14 | 0.98 | 0.84 | 0.87 | **1.40** | 1.00 |
| 37 | 51 | 0.96 | 1.01 | 1.06 | 1.00 | **1.28** | **1.29** | **0.19** | 1.06 | 0.94 | 0.89 | **1.25** | 0.96 | 0.90 | 0.86 | **1.30** | 0.99 |
| 38 | 53 | 0.94 | 1.01 | 1.04 | 1.04 | **1.34** | **1.26** | **0.23** | 0.96 | 0.94 | 0.88 | **1.25** | 0.98 | 0.88 | 0.90 | **1.30** | 0.99 |
| 39 | 54 | 0.97 | 1.06 | 0.99 | 1.01 | **1.40** | 1.12 | **0.27** | 0.98 | 0.94 | 0.94 | 1.17 | 1.00 | 0.82 | 0.90 | **1.38** | 1.01 |
| 40 | 55 | 0.95 | 0.99 | 1.06 | 1.03 | **1.32** | **1.24** | **0.22** | 1.01 | 1.00 | 0.96 | 1.08 | 0.98 | 0.86 | 0.89 | **1.38** | 0.98 |
| 41 | 56 | 0.97 | 1.04 | 1.04 | 0.99 | **1.35** | **1.20** | **0.21** | 1.01 | 0.94 | 0.98 | 1.13 | 0.99 | 0.86 | 0.85 | **1.38** | 1.01 |
| 42 | 57 | 0.95 | 1.03 | 1.06 | 1.00 | **1.36** | **1.21** | **0.21** | 0.98 | 0.95 | 0.96 | 1.09 | 1.03 | 0.86 | 0.87 | **1.37** | 1.00 |
| 43 | 58 | 0.97 | 1.00 | 1.05 | 1.00 | **1.29** | **1.21** | **0.26** | 1.05 | 0.99 | 0.95 | 1.12 | 0.96 | 0.86 | 0.89 | **1.34** | 0.99 |
| 44 | 59 | 0.95 | 1.03 | 1.04 | 1.01 | **1.33** | **1.24** | **0.23** | 0.99 | 0.95 | 0.94 | 1.15 | 1.00 | 0.87 | 0.85 | **1.36** | 1.00 |
| 45 | 60 | 0.97 | 1.00 | 1.00 | 1.04 | **1.35** | **1.25** | **0.27** | 0.94 | 0.95 | 0.97 | 1.15 | 0.98 | 0.85 | 0.87 | **1.37** | 1.01 |
| 46 | 61 | 0.98 | 1.04 | 0.98 | 1.01 | **1.33** | **1.24** | **0.28** | 0.96 | 0.96 | 0.92 | 1.16 | 0.99 | 0.83 | 0.85 | **1.39** | 1.03 |
| 47 | 62 | 0.96 | 1.07 | 1.02 | 0.99 | **1.27** | **1.26** | **0.30** | 1.00 | 0.97 | 0.98 | 1.13 | 0.96 | 0.90 | **0.77** | **1.31** | 1.04 |
| 48 | 63 | 0.97 | 1.03 | 1.05 | 0.97 | **1.30** | **1.21** | **0.24** | 1.04 | 0.94 | 0.94 | 1.10 | 1.04 | 0.87 | 0.87 | **1.37** | 0.97 |
| 49 | 64 | 0.95 | 1.04 | 1.00 | 1.03 | **1.36** | **1.25** | **0.29** | 0.92 | 0.98 | 0.97 | 1.11 | 0.97 | 0.83 | **0.79** | **1.41** | 1.05 |
| 50 | 66 | 0.95 | 1.01 | 1.04 | 1.03 | **1.35** | 1.19 | **0.28** | 0.96 | 0.95 | 1.00 | 1.11 | 0.99 | 0.86 | 0.87 | **1.36** | 1.00 |
| 51 | 67 | 0.92 | 1.04 | 1.03 | 1.05 | **1.34** | **1.26** | **0.29** | 0.94 | 0.96 | 0.96 | 1.16 | 0.96 | 0.90 | **0.80** | **1.32** | 1.01 |
| 52 | 68 | 0.94 | 1.04 | 1.04 | 1.02 | **1.37** | **1.22** | **0.20** | 0.96 | 0.92 | 0.92 | **1.21** | 1.00 | 0.87 | 0.85 | **1.37** | 1.00 |
| 53 | 7 | 0.96 | 1.00 | 1.05 | 1.01 | **1.29** | **1.24** | **0.31** | 0.99 | 1.00 | 0.92 | 1.09 | 0.99 | 0.86 | 0.91 | **1.32** | 1.00 |
| 54 | 71 | 0.94 | 1.01 | 1.07 | 1.02 | **1.30** | **1.26** | **0.26** | 0.99 | 0.97 | 0.87 | 1.19 | 0.99 | 0.88 | 0.91 | **1.30** | 0.99 |
| 55 | 65 | 0.95 | 1.02 | 1.03 | 1.03 | **1.32** | **1.25** | **0.25** | 0.99 | 0.95 | 0.93 | **1.21** | 0.96 | 0.88 | 0.86 | **1.33** | 1.00 |
| 56 | 73 | 0.95 | 1.01 | 0.98 | 1.06 | **1.27** | **1.27** | **0.30** | 1.00 | 1.00 | 0.96 | 1.13 | 0.94 | 0.87 | 0.83 | **1.40** | 0.97 |
| 57 | 74 | 0.95 | 1.04 | 1.03 | 1.01 | **1.35** | **1.25** | **0.24** | 0.97 | 0.99 | 0.98 | 1.12 | 0.94 | 0.84 | **0.80** | **1.38** | 1.05 |
| 58 | 75 | 0.98 | 0.98 | 0.97 | 1.06 | **1.31** | **1.35** | **0.27** | 0.92 | 0.97 | 0.97 | 1.19 | 0.92 | 0.84 | 0.81 | **1.39** | 1.04 |
| 59 | 76 | 0.96 | 1.00 | 1.06 | 1.01 | **1.32** | **1.24** | **0.22** | 1.02 | 0.96 | 0.97 | 1.08 | 1.01 | 0.86 | 0.86 | **1.40** | 0.97 |
| 60 | 77 | 0.92 | 1.02 | 1.06 | 1.04 | **1.37** | **1.21** | **0.25** | 0.97 | 0.98 | 1.01 | 1.11 | 0.95 | 0.88 | 0.84 | **1.32** | 1.01 |
| 61 | 78 | 0.93 | 0.99 | 1.11 | 1.02 | **1.36** | **1.26** | **0.21** | 0.96 | 1.00 | 0.94 | 1.10 | 0.97 | 0.84 | 0.87 | **1.35** | 1.03 |
| 62 | 8 | 0.90 | 1.04 | 1.05 | 1.05 | **1.39** | **1.25** | **0.26** | 0.89 | 0.96 | 0.92 | 1.19 | 0.98 | 0.87 | 0.83 | **1.33** | 1.04 |
| 63 | 80 | 0.93 | 1.03 | 1.08 | 1.01 | **1.37** | **1.23** | **0.20** | 0.97 | 0.95 | 0.89 | 1.19 | 1.00 | 0.88 | 0.88 | **1.30** | 1.01 |
| 64 | 81 | 0.93 | 1.07 | 1.00 | 1.04 | **1.40** | **1.20** | **0.19** | 0.96 | 0.95 | 0.91 | 1.17 | 1.00 | 0.85 | 0.85 | **1.42** | 0.98 |
| 65 | 82 | 0.93 | 0.99 | 1.04 | 1.06 | **1.31** | **1.26** | **0.28** | 0.99 | 1.01 | 0.99 | 1.10 | 0.93 | 0.87 | 0.85 | **1.36** | 0.99 |
| 66 | 85 | 0.97 | 1.04 | 1.00 | 1.00 | **1.34** | **1.23** | **0.24** | 0.99 | 0.97 | 0.96 | 1.14 | 0.96 | 0.82 | 0.83 | **1.40** | 1.03 |
| 67 | 88 | 1.01 | 1.01 | 1.00 | 0.99 | **1.25** | 1.18 | **0.30** | 1.08 | 0.97 | 0.95 | 1.15 | 0.97 | 0.85 | 0.91 | **1.36** | 0.98 |
| 68 | 89 | 0.97 | 1.01 | 1.06 | 0.99 | **1.26** | **1.26** | **0.25** | 1.06 | 0.99 | 0.88 | 1.11 | 1.01 | 0.87 | 0.90 | **1.35** | 0.97 |
| 69 | 9 | 0.96 | 1.03 | 1.03 | 1.06 | **1.30** | **1.22** | **0.26** | 0.96 | 1.02 | 0.92 | 1.12 | 0.94 | 0.89 | **0.79** | **1.38** | 0.99 |
| 70 | 90 | 0.99 | 1.03 | 1.01 | 1.00 | **1.35** | **1.24** | **0.23** | 0.96 | 0.96 | 0.97 | 1.19 | 1.01 | 0.83 | 0.84 | **1.42** | 0.95 |
| 71 | 94 | 0.92 | 1.03 | 1.00 | 1.05 | **1.42** | **1.33** | **0.31** | 0.97 | 0.97 | 1.02 | 1.08 | 0.93 | 0.81 | 0.91 | **1.37** | 0.94 |
| 72 | 95 | 0.93 | 1.13 | 1.05 | 1.02 | **1.51** | **1.41** | **0.25** | 0.90 | 0.96 | 0.95 | 1.15 | 0.92 | 0.84 | 0.84 | **1.26** | 0.94 |
| 73 | 96 | 0.96 | 1.04 | 1.01 | 1.02 | **1.30** | **1.29** | **0.29** | 0.98 | 1.01 | 0.87 | 1.14 | 0.97 | 0.84 | 0.86 | **1.36** | 1.01 |
| 74 | 98 | 0.96 | 1.06 | 1.01 | 1.01 | **1.39** | 1.19 | **0.23** | 0.94 | 0.95 | 0.95 | 1.15 | 0.99 | 0.84 | 0.85 | **1.37** | 1.03 |
| 75 | 31 | 0.98 | 1.01 | 0.99 | 1.02 | **1.38** | **1.20** | **0.29** | 0.93 | 0.92 | 1.03 | 1.15 | 0.97 | 0.84 | 0.84 | **1.36** | 1.03 |
|  | Mean A | 0.96 | 1.02 | 1.02 | 1.02 | 1.34 | **1.23** | 0.25 | 0.98 | 0.97 | 0.96 | 1.14 | 0.97 | 0.85 | 0.85 | **1.37** | 1.01 |
| 76 | 14 | 0.97 | 1.06 | 1.03 | 0.98 | **1.38** | 1.17 | **0.30** | 0.95 | 0.97 | 0.82 | **1.20** | 1.02 | **0.78** | 0.94 | **1.34** | 1.05 |
| 77 | 17 | 0.98 | 1.06 | 1.02 | 0.96 | **1.33** | 1.14 | **0.34** | 1.00 | 0.96 | 0.82 | **1.20** | 1.03 | **0.79** | 0.96 | **1.34** | 1.02 |
| 78 | 26 | 0.96 | 1.05 | 1.02 | 1.00 | **1.41** | **1.27** | **0.23** | 0.86 | 0.97 | 0.83 | 1.18 | 1.03 | **0.79** | 0.86 | **1.38** | 1.08 |
| 79 | 27 | 0.93 | 1.08 | 1.07 | 0.98 | **1.40** | **1.23** | **0.26** | 0.90 | 0.98 | 0.84 | 1.19 | 1.00 | 0.82 | 0.86 | **1.30** | 1.10 |
| 80 | 31 | 0.91 | 1.09 | 1.11 | 0.96 | **1.38** | **1.20** | **0.26** | 0.95 | 1.00 | 0.85 | 1.18 | 0.99 | 0.83 | 0.86 | **1.28** | 1.09 |
| 81 | 35 | 0.94 | 1.08 | 1.05 | 0.98 | **1.37** | **1.27** | **0.28** | 0.90 | 0.98 | 0.79 | 1.17 | 1.05 | 0.82 | 0.87 | **1.34** | 1.06 |
| 82 | 37 | 0.98 | 1.06 | 1.03 | 0.96 | **1.32** | **1.30** | **0.26** | 0.91 | 0.97 | 0.82 | 1.19 | 1.03 | **0.80** | 0.83 | **1.37** | 1.09 |
| 83 | 4 | 0.96 | 1.05 | 1.08 | 0.95 | **1.40** | **1.23** | **0.22** | 0.91 | 0.94 | 0.87 | **1.21** | 1.01 | **0.80** | 0.87 | **1.30** | 1.11 |
| 84 | 42 | 0.95 | 1.11 | 1.05 | 0.95 | **1.35** | **1.22** | **0.26** | 0.94 | 0.97 | 0.85 | 1.16 | 1.01 | 0.83 | 0.82 | **1.34** | 1.09 |
| 85 | 48 | 0.95 | 1.06 | 1.07 | 0.96 | **1.35** | 1.19 | **0.29** | 0.95 | 0.98 | 0.85 | 1.19 | 1.00 | 0.81 | 0.89 | **1.31** | 1.09 |
| 86 | 5 | 0.95 | 1.06 | 1.07 | 0.97 | **1.40** | **1.24** | **0.22** | 0.90 | 0.93 | 0.91 | **1.20** | 1.00 | 0.83 | 0.82 | **1.31** | 1.10 |
| 87 | 52 | 0.96 | 1.09 | 1.07 | 0.93 | **1.35** | 1.19 | **0.32** | 0.95 | 0.96 | 0.84 | 1.18 | 1.03 | **0.80** | 0.87 | **1.32** | 1.09 |
| 88 | 6 | 0.96 | 1.07 | 1.11 | 0.92 | **1.36** | **1.20** | **0.25** | 0.97 | 0.94 | 0.85 | 1.16 | 1.07 | 0.84 | 0.88 | **1.30** | 1.06 |
| 89 | 69 | 0.95 | 1.06 | 1.08 | 0.96 | **1.39** | 1.18 | **0.31** | 0.93 | 0.94 | 0.90 | **1.22** | 0.98 | 0.81 | 0.86 | **1.27** | 1.13 |
| 90 | 70 | 0.96 | 1.04 | 1.05 | 0.98 | **1.33** | 1.15 | **0.31** | 1.00 | 0.94 | 0.84 | **1.22** | 1.03 | 0.84 | 0.96 | **1.31** | 0.99 |
| 91 | 72 | 0.99 | 1.07 | 1.02 | 0.95 | **1.37** | **1.24** | **0.28** | 0.91 | 0.94 | 0.82 | **1.20** | 1.06 | **0.78** | 0.87 | **1.36** | 1.08 |
| 92 | 79 | 0.93 | 1.03 | 1.08 | 1.00 | **1.37** | **1.30** | **0.25** | 0.89 | 1.01 | 0.86 | 1.18 | 0.97 | 0.82 | 0.85 | **1.32** | 1.09 |
| 93 | 83 | 0.96 | 1.09 | 1.02 | 0.98 | **1.38** | **1.23** | **0.28** | 0.92 | 0.97 | 0.80 | **1.25** | 0.99 | 0.82 | 0.88 | **1.29** | 1.09 |
| 94 | 84 | 0.96 | 1.03 | 1.10 | 0.96 | **1.39** | **1.24** | **0.19** | 0.94 | 0.94 | 0.86 | **1.21** | 1.02 | 0.81 | 0.90 | **1.31** | 1.07 |
| 95 | 86 | 0.97 | 1.01 | 1.07 | 0.98 | **1.34** | **1.27** | **0.21** | 0.97 | 1.01 | 0.85 | 1.17 | 0.97 | 0.81 | 0.91 | **1.33** | 1.06 |
| 96 | 91 | 0.95 | 1.05 | 1.06 | 0.97 | **1.35** | 1.17 | **0.35** | 0.92 | 0.95 | 0.86 | **1.21** | 1.02 | 0.82 | 0.91 | **1.28** | 1.08 |
| 97 | 92 | 0.95 | 1.01 | 1.11 | 0.97 | **1.36** | **1.32** | **0.22** | 0.92 | 1.00 | 0.84 | 1.11 | 1.04 | **0.80** | 0.87 | **1.36** | 1.06 |
| 98 | 93 | 0.92 | 1.07 | 1.05 | 1.01 | **1.39** | **1.20** | **0.24** | 0.94 | 0.96 | 0.88 | **1.22** | 0.99 | 0.85 | 0.87 | **1.33** | 1.03 |
| 99 | 97 | 0.96 | 1.04 | 1.02 | 1.01 | **1.40** | **1.22** | **0.27** | 0.88 | 0.94 | 0.86 | **1.23** | 1.00 | **0.80** | 0.90 | **1.34** | 1.07 |
| 100 | 99 | 0.97 | 1.06 | 1.03 | 0.97 | **1.42** | 1.16 | **0.26** | 0.90 | 0.93 | 0.90 | **1.21** | 1.01 | **0.79** | 0.89 | **1.33** | 1.09 |
|  | Mean B | 0.95 | 1.06 | 1.06 | 0.97 | **1.37** | **1.22** | **0.27** | 0.93 | 0.96 | 0.85 | 1.19 | 1.01 | 0.81 | 0.88 | **1.32** | 1.08 |
| 101 | NAT001 | 0.96 | 1.07 | 1.01 | 0.98 | **1.33** | 1.17 | **0.30** | 1.05 | 1.02 | 0.86 | 1.09 | 1.01 | **0.75** | 0.88 | **1.51** | 0.97 |
| 102 | NAT045 | 0.94 | 1.10 | 1.04 | 0.96 | **1.33** | **1.23** | **0.29** | 1.00 | 0.92 | 0.84 | **1.23** | 1.04 | 0.86 | 0.82 | **1.37** | 1.02 |
| 103 | QPM | 0.96 | 1.11 | 1.00 | 0.96 | **1.35** | 1.16 | **0.27** | 1.03 | 0.98 | 0.84 | 1.15 | 1.04 | **0.77** | 0.86 | **1.51** | 0.99 |
| 104 | C024 | 0.94 | 1.13 | 1.01 | 0.95 | **1.32** | 1.15 | **0.31** | 1.05 | 0.92 | 0.89 | 1.19 | 0.97 | 0.87 | **0.80** | **1.42** | 0.99 |
| 105 | C025 | 0.92 | 1.19 | 1.00 | 0.96 | **1.35** | 1.12 | **0.27** | 1.06 | 0.93 | 0.91 | 1.18 | 1.02 | 0.88 | **0.75** | **1.45** | 0.98 |
| 106 | C026 | 0.93 | 1.09 | 1.01 | 0.99 | **1.39** | 1.15 | **0.30** | 0.97 | 0.99 | 0.83 | 1.14 | 1.04 | **0.75** | 0.90 | **1.47** | 1.00 |
| 107 | NY-074 | 0.94 | 1.07 | 1.04 | 0.99 | **1.34** | 1.12 | **0.31** | 1.06 | 0.95 | 0.96 | 1.11 | 0.97 | 0.82 | 0.84 | **1.43** | 0.98 |
| 108 | N4 | 0.90 | 1.11 | 0.98 | 1.04 | **1.37** | 1.12 | **0.28** | 0.97 | 0.89 | 0.81 | **1.37** | 1.00 | 0.90 | 0.87 | **1.33** | 0.97 |
| 109 | N10 | 0.99 | 1.04 | 0.93 | 1.04 | **1.26** | **1.24** | **0.38** | 0.98 | 0.94 | 0.83 | **1.26** | 1.00 | 0.84 | 0.89 | **1.39** | 0.97 |
| 110 | QCE | 0.96 | 1.09 | 0.98 | 0.98 | **1.42** | 1.12 | **0.33** | 0.93 | 0.98 | 0.92 | 1.13 | 0.99 | **0.69** | 0.84 | **1.52** | 1.09 |
| 111 | CL-170085 | 0.90 | 1.15 | 1.03 | 0.98 | **1.36** | 1.15 | **0.29** | 1.01 | 0.94 | 0.92 | 1.12 | 1.05 | 0.89 | **0.76** | **1.43** | 0.98 |
|  | Mean C | 0.94 | 1.10 | 1.00 | 0.98 | **1.35** | 1.16 | **0.30** | 1.01 | 0.95 | 0.87 | 1.18 | 1.01 | 0.82 | 0.84 | **1.44** | 0.99 |
