# Supplementary figures and images for: The Genomic Signature of Human Rhinoviruses A, B and C
Source: PLoS One. 2012 Sep 13;7(9):e44557. doi: 10.1371/journal.pone.0044557 (PMC3441561; doi:10.1371/journal.pone.0044557)

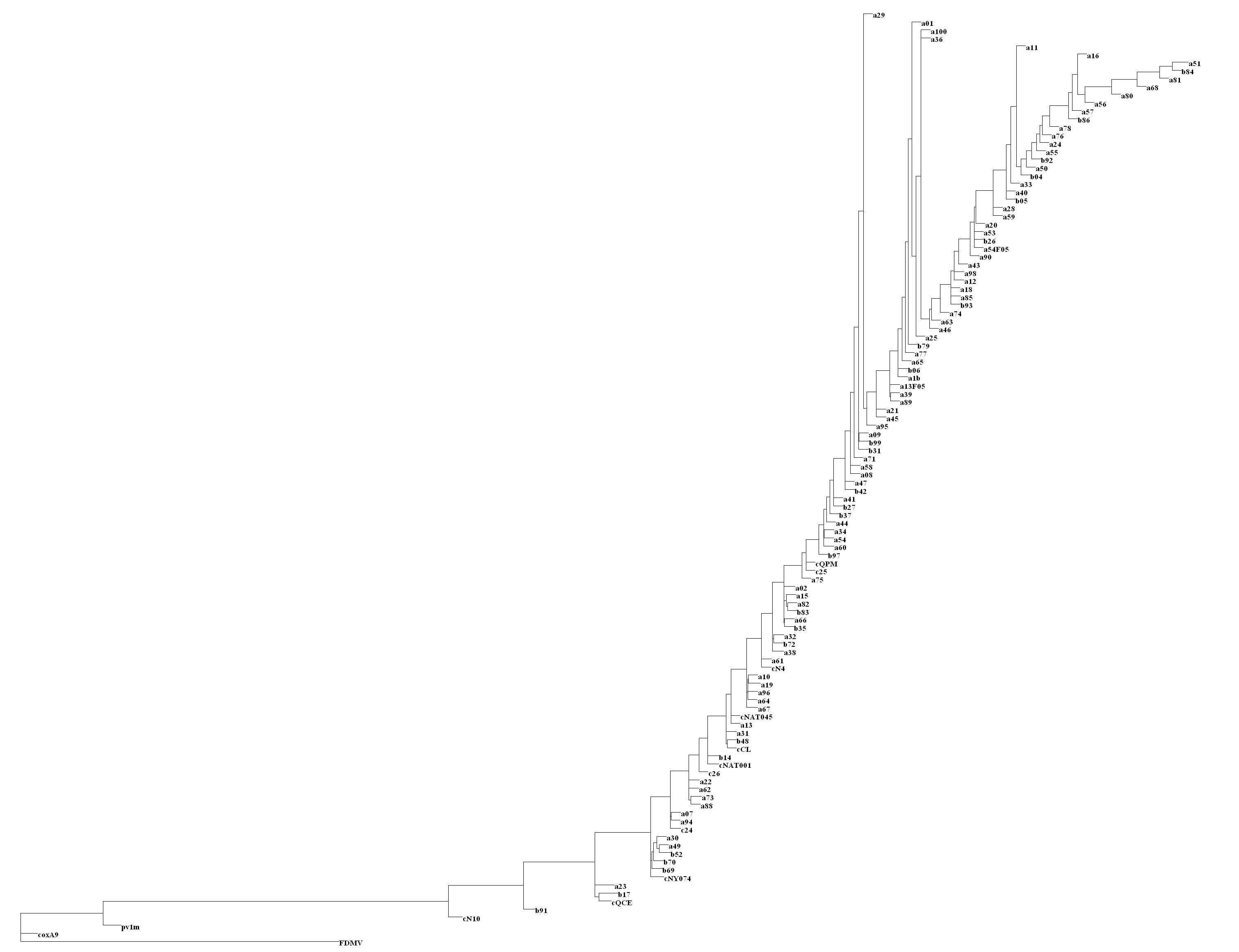

Supplement: Figure S1 — Pairwise distance tree based on RCpG values constructed using the FastME algorithm. (TIF) [file pone.0044557.s002.tif]

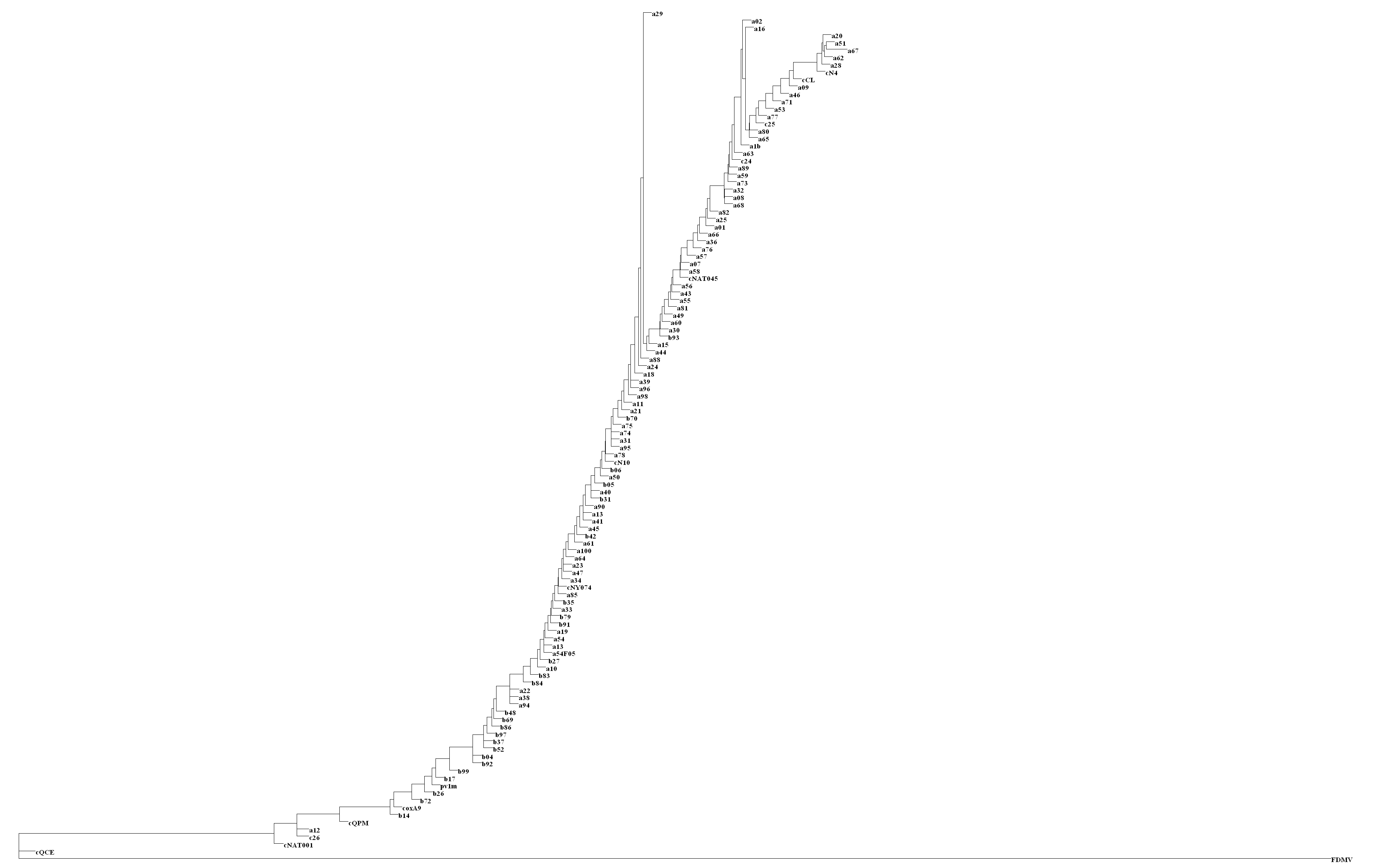

Supplement: Figure S2 — Pairwise distance tree based on RUpA values values constructed using the FastME algorithm. (TIF) [file pone.0044557.s003.tif]
